# Supplementary material for: In Vitro Effect of Three-Antibiotic Combinations plus Potential Antibiofilm Agents against Biofilm-Producing Mycobacterium avium and Mycobacterium intracellulare Clinical Isolates
Source: Antibiotics (Basel). 2023 Sep 6;12(9):1409. doi: 10.3390/antibiotics12091409 (PMC10526108; doi:10.3390/antibiotics12091409)
Supplement: Supplementary file 1 [file antibiotics-12-01409-s001.zip › antibiotics-2570201-supplementary.pdf]

Supplementary Materials

Table S1: Individual MICs of the antibiotics alone in in MAC clinical isolates.

	MIC (µg/mL)							
**Antibiotic**	***M. intracellulare***				***M. avium***			
	M. intracellulare 1	*M. intracellulare* 2	*M. intracellulare* 3	*M. intracellulare* 4	*M. avium* 1	*M. avium* 2	*M. avium* 3	*M. avium* 4
**CLA**	8	8	2	2	4	2	4	<0.25
**MOX**	16	2	2	4	4	4	4	<0.25
**BEDA**	2	2	0.5	0.5	0.5	0.5	0.5	1
**CLO**	16	4	16	16	4	2	4	4
**RB**	0.25	0.25	0.25	0.5	<0.25	4	0.25	1
**RF**	0.5	0.25	0.5	0.5	<0.25	2	0.25	<0.25
**EB**	32	32	2	2	8	8	8	8

MIC: minimum inhibitory concentration (µg/mL), MAC: *Mycobacterium avium* complex, CLA: clarithromycin, MOX: moxifloxacin, BED: bedaquiline, CLO: clofazimine, RB: rifabutin, RIF: rifampicin, EMB: ethambutol
